# Endoscopically Assisted Management of Bilateral Coronoid Hyperplasia: A Case Report with Oral-Health-Related Quality of Life Outcomes

**DOI:** 10.3390/jcm15176591

**Published:** 2026-08-26

**Authors:** Juraj Brozović, Igor Čvrljević, Marko Tarle, Barbara Mikulić, Bruno Vidaković, Ivica Lukšić

**Affiliations:** 1 Faculty of Dental Medicine and Health, University of Osijek, 31000 Osijek, Croatia; barbaramikulic@ymail.com; 2 Department of Maxillofacial and Oral Surgery, Dubrava University Hospital, 10000 Zagreb, Croatia; igor.cvrljevic@gmail.com (I.Č.); mtarle@sfzg.hr (M.T.); luksic@kbd.hr (I.L.); 3 School of Medicine, University of Zagreb, 10000 Zagreb, Croatia; 4 School of Dental Medicine, University of Zagreb, 10000 Zagreb, Croatia

**Keywords:** trismus, mandibular diseases, endoscopy, quality of life, visual analogue scale

## Abstract

**Background/Objectives:** This case report describes a rare case of bilateral coronoid hyperplasia (CPH) with suspected Jacob’s disease in a young female patient, managed by endoscopically assisted coronoidectomy, and provides quantitative oral-health-related quality of life (OHRQoL) and pain outcomes. **Methods:** An 18-year-old female with severely restricted mouth opening underwent bilateral endoscopically assisted intraoral coronoidectomy. OHRQoL and pain were assessed with the Oral Health Impact Profile 14 (OHIP-CRO14) and a 100 mm visual analogue scale (VAS) preoperatively, daily for seven postoperative days, and at one year. **Results:** Maximum mouth opening improved from 8 mm to 35 mm at nine months (+338%). OHIP-14 total scores remained within a “poor”-to-“moderate” impact and low quality of life from the preoperative value (38) throughout the postoperative week (31–38) and decreased to 14 at one year (−63%; “good” impact, very high quality of life). Psychological disability and psychological discomfort were the most affected domains. VAS scores rose from 36 preoperatively, peaked at 55 on day 2, fluctuated as mild pain (27–44), and resolved at one year. **Conclusions:** Endoscopically assisted intraoral coronoidectomy restored functional mouth opening and produced clinically meaningful improvements in pain and OHRQoL, with the largest gains in psychological domains. To the authors’ knowledge, this is the first case report providing quantified, domain-resolved OHRQoL outcomes after coronoidectomy.

## 1. Introduction

Coronoid process hyperplasia (CPH) is a rare mandibular developmental disorder characterized by an abnormally enlarged coronoid process, causing painless and progressive mechanical limitation of mouth opening [1]. Its underlying nature is truly hyperplastic, where the zygomatically impinging portion is composed of histologically normal bone, also termed Langenbeck disease. CPH mostly affects men (male-to-female ratio 5:1) from adolescence into adulthood; however, the scarcity of epidemiological studies makes it difficult to establish its true incidence and prevalence [2]. A sample-limited study noted a prevalence of 0.3% [3], whereas other reports found that up to 5% of limited mouth-opening cases were attributable to elongation of the coronoid process [4]. Such cases were mostly bilateral, with a mean maximal mouth opening of 16.5 mm at diagnosis [5,6]. The attempts at clarifying the disorder’s etiology included the following theories: temporal muscle hyperactivity, temporomandibular joint disorders (TMDs), facial trauma, hormonal alterations, hereditary traits, and a rare autosomal dominant disorder—trismus-pseudocamptodactyly syndrome (TPS). In addition to coronoid hyperplasia, TPS features short stature, flexor tendon shortening (curving fingers) and foot deformities [7,8,9]. Further distinctive pathologies like fractures, osteoma or osteochondroma can result in similar symptoms, with the latter presenting a layer of cartilaginous tissue [10].

Jacob’s disease (JD) is defined by a rare pseudo-joint formation between the inner aspect of the malar bone and the coronoid process, retaining the process in a non-functional articulation. It can be a consequence of either CPH, osteochondroma or any chronic impingement of articulating surfaces involved [10,11]. A recently proposed classification of JD denotes the mildest involvement as Grade 1, where ipsi- or bilateral pseudojoint is formed between an abnormal coronoid process and zygomatic bone, with maximum mouth opening (MMO) greater than or equal to 20 mm. Further aggravation, with MMO between 10 and 20 mm is classified as Grade 2, and the gravest involvement with 10 mm or less as Grade 3 [1].

The diagnosis of CPH and JD is often considered only after more common causes of limited mouth opening have been excluded: intracapsular, such as disc displacement without reduction and osteoarthritis, and extracapsular masticatory muscle disorders. These disorders usually improve with conservative treatments—counselling, physical therapy, medication and occlusal splints [12,13]. Where ineffective, further diagnostic modalities, like computed tomograms, visualize the articular region and facilitate accurate diagnosis and possible surgical indications [14].

In cases where the coronoid process extends more than 1 cm above the inferior aspect of the zygomatic arch and limits the opening and lateral excursive movements, and when a pseudojoint is termed Grade 2 and 3, non-surgical treatments lead to limited or no improvement. Thus, surgical intervention is generally indicated [1,15,16]. Procedurally, the abnormal coronoid process may be detached from the ramus (coronoidotomy) or completely resected (coronoidectomy) by an intra- or extraoral approach. The intraoral approach is potentially less invasive, particularly in terms of avoiding facial nerve damage and skin scarring. Recent reports emphasize the key role of the endoscope in visualizing the poorly visible surgical field and reducing postoperative sequelae [9,16,17,18].

The detrimental effects of CPH and JD severely impact patients’ quality of life, and their surgical management leads to significant improvements [16,19]. To the best of the authors’ knowledge, there has been no report on measurable oral-health-related quality of life (OHRQoL) outcomes after coronoidectomy for restricted mouth opening in the literature to date. Here we describe a rare case of bilateral coronoid hyperplasia with suspected Jacob’s disease in a young female patient, managed by endoscopically assisted intraoral bilateral coronoidectomy, and we report—to the best of the authors’ knowledge, for the first time—quantitative patient-reported quality of life and pain outcomes preoperatively, during the immediate postoperative period, and at the one-year recall. The manuscript is reported in accordance with the CARE guideline; the completed checklist is included (Table S1). The conduct of the case followed the ethical principles of the Declaration of Helsinki and institutional requirements, as verified by the Ethics Committee of Dubrava University Hospital (ref. 2026/0521-4).

## 2. Case Presentation

### 2.1. Case History

**Figure 1:**
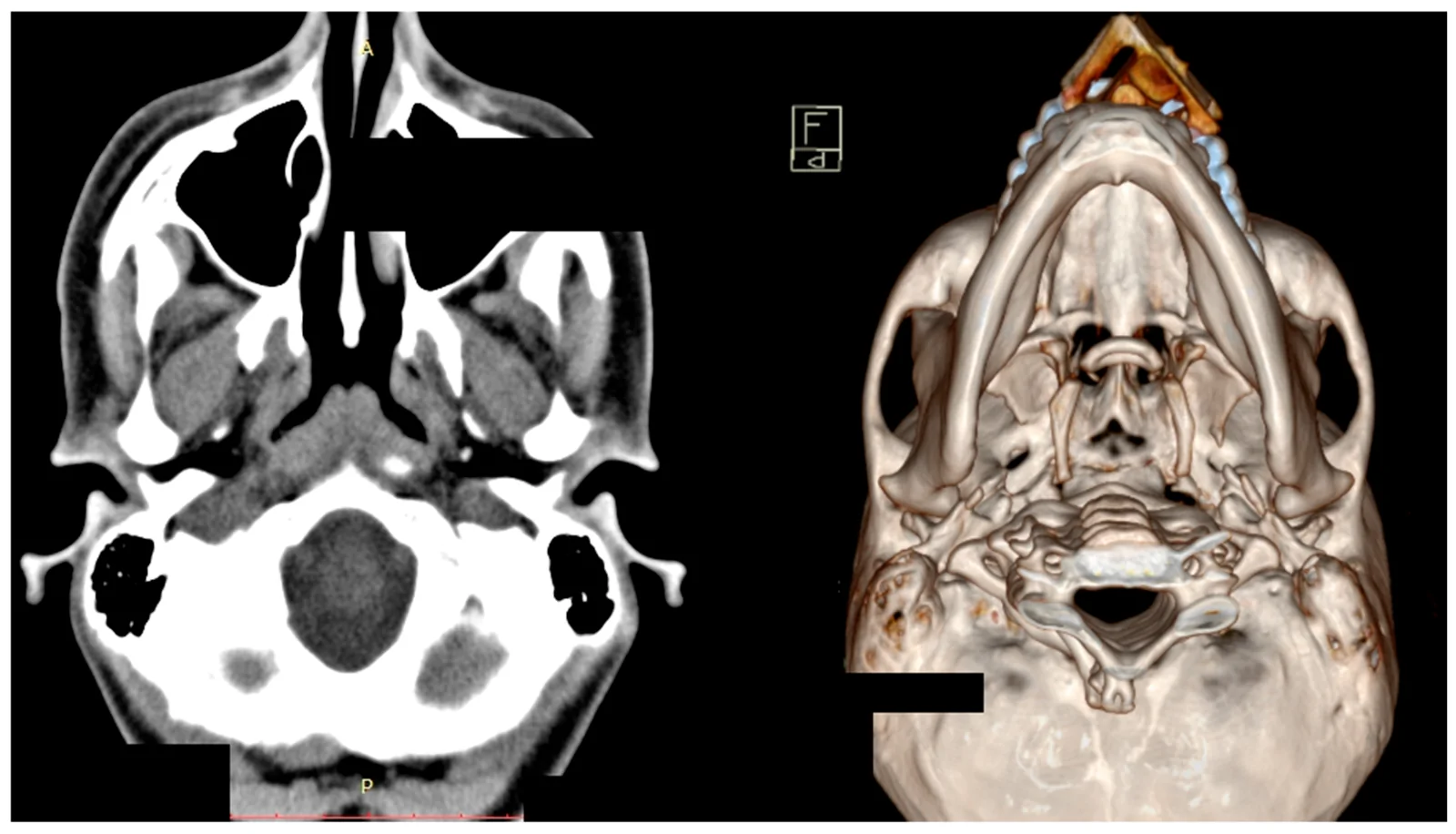
Preoperative MSCT at the age of 13. (**Left**): Axial slice at the level of the skull base and zygomatic arches, showing bilaterally enlarged coronoid processes in contact with the temporal surfaces of the zygomatic bones. (**Right**): Submental three-dimensional render view, demonstrating the hyperplastic, stumpy coronoid processes bilaterally with remodelling at the zygomatic contact areas.

A 17-year-old female patient (aged 18 at surgery) was admitted to our oral surgery clinic with complaints of severe limitation in mouth opening. This admission was the authors’ first contact with the patient; the earlier findings summarized below were reconstructed from the previous medical records. The limitation was first noted at the age of 12, and had been steadily advancing over the years to the point of near-full jaw lock, which significantly impacted her quality of life. The clinical course, investigations, surgical treatment, rehabilitation and follow-up are summarized in Table 1. Her medical history revealed uneventful birth, and normal growth and psychomotor development, although described as petite at the age of 14 (W 45 kg, H 156 cm), with mild face asymmetry (right side larger), subtle right eyelid ptosis, planovalgus foot and metatarsus adductus. Due to aggravation of limited mouth opening, she had previously undergone magnetic resonance imaging (MRI) and multislice computed tomography (MSCT) of the oral and maxillofacial region at the age of 13. The MRI showed no temporomandibular joint pathology, while the MSCT finding revealed hyperplastic, voluminous and stumpy coronoid processes bilaterally, with apparent remodelling upon repeated contact with zygomatic bone (Figure 1).

On the zygomatic side, medial and inferior aspects exhibited tumefactive, mildly exophytic borders, protruding posteromedially. The tips of coronoid processes at the time extended beyond the level of the zygomatic arch by 3 mm in a closed-mouth position. A comparable diagnosis had reportedly been established in her father, although without formal documentation. A raised suspicion of trismus-pseudocamptodactyly syndrome prompted genetic testing at the age of 14 at the Laboratory for Medical Genetics (Children’s Hospital Zagreb, Croatia), comprising clinical exome sequencing with phenotype-driven gene panels covering trismus (including the myosin heavy chain 8 gene, MYH8), short stature, relative macrocephaly, facial asymmetry, unilateral ptosis, planovalgus feet, metatarsus adductus and arthrogryposis, together with chromosomal microarray analysis (CMA). No clinically significant variants associated with the referral diagnosis were identified in the analyzed genes, and no pathogenic or likely pathogenic MYH8 variants were found, so trismus-pseudocamptodactyly syndrome was not confirmed. At the age of 15, she was examined by a physiatrist for tendon and joint pain in ankles, and the pain was conjecturally attributed to a growth spurt. There were no traumatic events reported.

### 2.2. Surgical and Postoperative Phase

**Figure 2:**
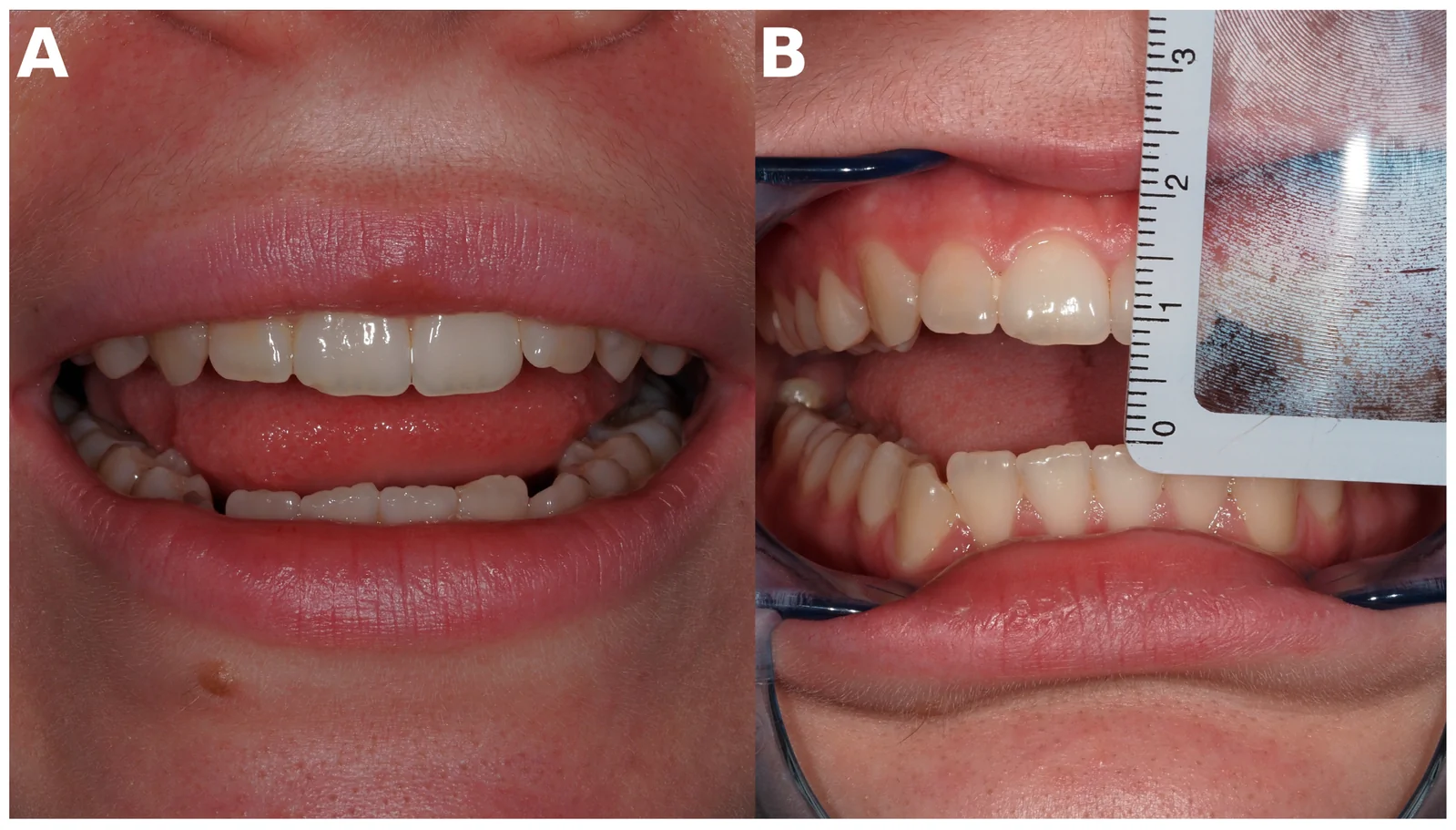
Maximal interincisal opening at the age of 17. (**A**) Frontal view at maximal unassisted opening; (**B**) lateral close-up with a millimetre ruler confirming an interincisal distance of 8 mm.

At admission, clinical examination revealed marked, mildly painful limitation of jaw movement, with a maximal interincisal opening of 8 mm (Figure 2), and no apparent deviation at the midpoint or deflection at the endpoint. Maximum mouth opening (MMO) was recorded as the maximal unassisted interincisal distance, measured between the incisal edges of the corresponding maxillary and mandibular central incisors. A digital calliper (DIN 862; Helios-Preisser, Gammertingen, Germany) was used, the reading was checked against a millimetre ruler, and values were rounded to the nearest millimetre. Formal MMO recordings were obtained at admission and at the one-month, nine-month and one-year follow-up visits, all performed by the same examiner. Assisted opening showed no improvement, with a hard-end feel upon further downward pressure. Lateral and protrusive movements were completely blocked. No joint sounds (clicking or crepitus) were detected. No palpable hypertrophy or tenderness of the masticatory muscles or temporomandibular joint was noted.

Intracapsular disorders such as disc displacement without reduction and osteoarthritis were therefore unlikely. There was no midline deviation or endpoint deflection, no joint sounds and no joint tenderness, and the end feel was hard with no gain on assisted opening. The masticatory muscles were neither tender nor hypertrophic, which argued against an extracapsular, muscle-related limitation [12,13]. In addition, the MRI of the temporomandibular joints performed at the age of 13 had shown no joint pathology.

**Figure 3:**
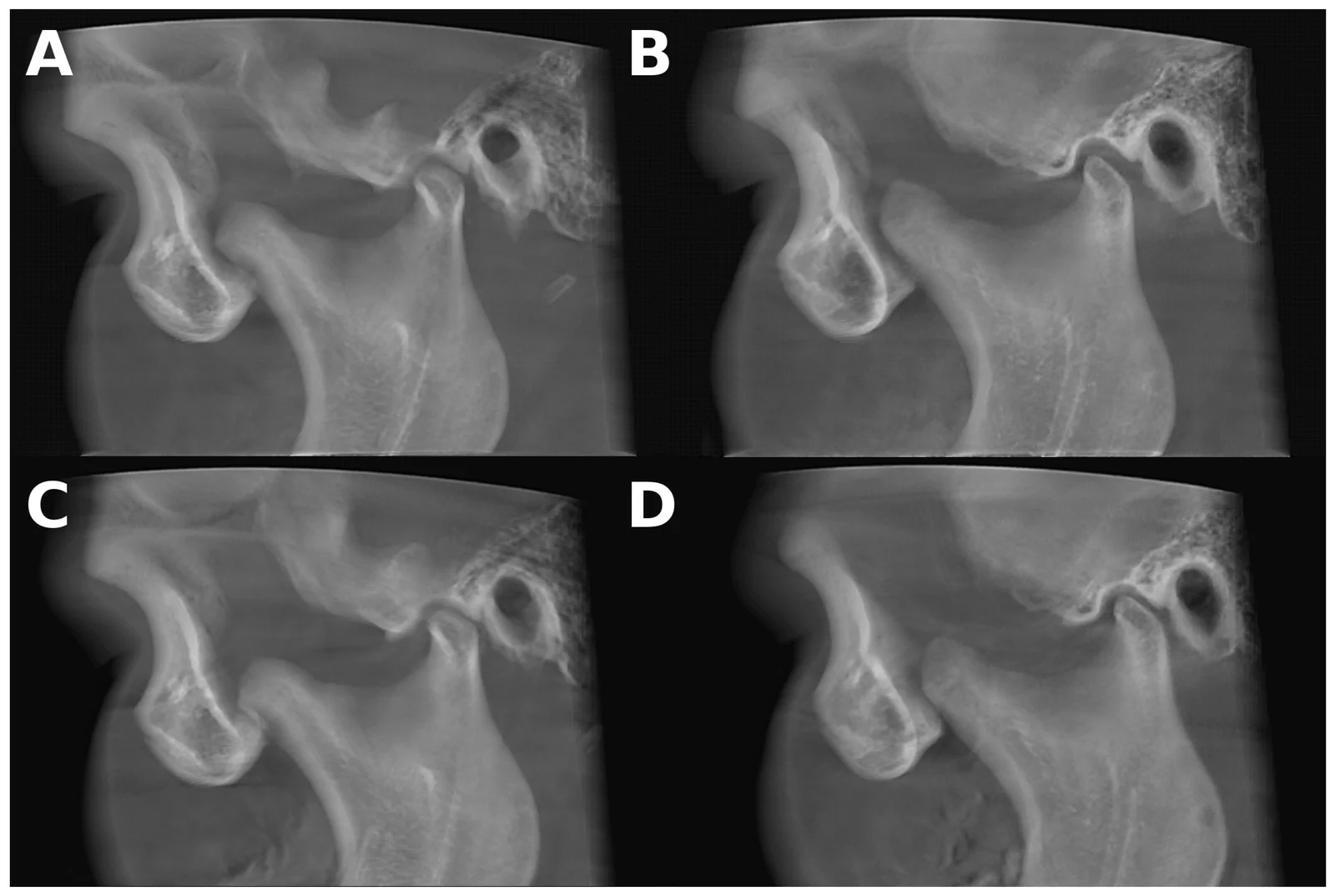
Preoperative CBCT, sagittal views of the ramus region. (**A**) Left side, closed mouth; (**B**) right side, closed mouth; (**C**) left side, open mouth; (**D**) right side, open mouth. In all views, the elongated coronoid process remains superimposed on the zygomatic bone, with minimal positional change between closed- and open-mouth acquisitions.

Pre-operative cone-beam computed tomography (CBCT), obtained shortly before the patient turned 18, confirmed the previous radiographic findings (Figure 3) and additionally suggested fibrous adhesions between the coronoid processes and the temporal surfaces of the zygomatic bones. Together with the earlier MSCT signs of mutual coronoid–zygomatic remodelling with tumefactive, mildly exophytic zygomatic borders, these radiological findings raised the suspicion of an accompanying pseudo-joint; given the MMO below 10 mm, a provisional diagnosis of Grade 3 JD according to the classification of Raccampo et al. [1] was made.

**Figure 4:**
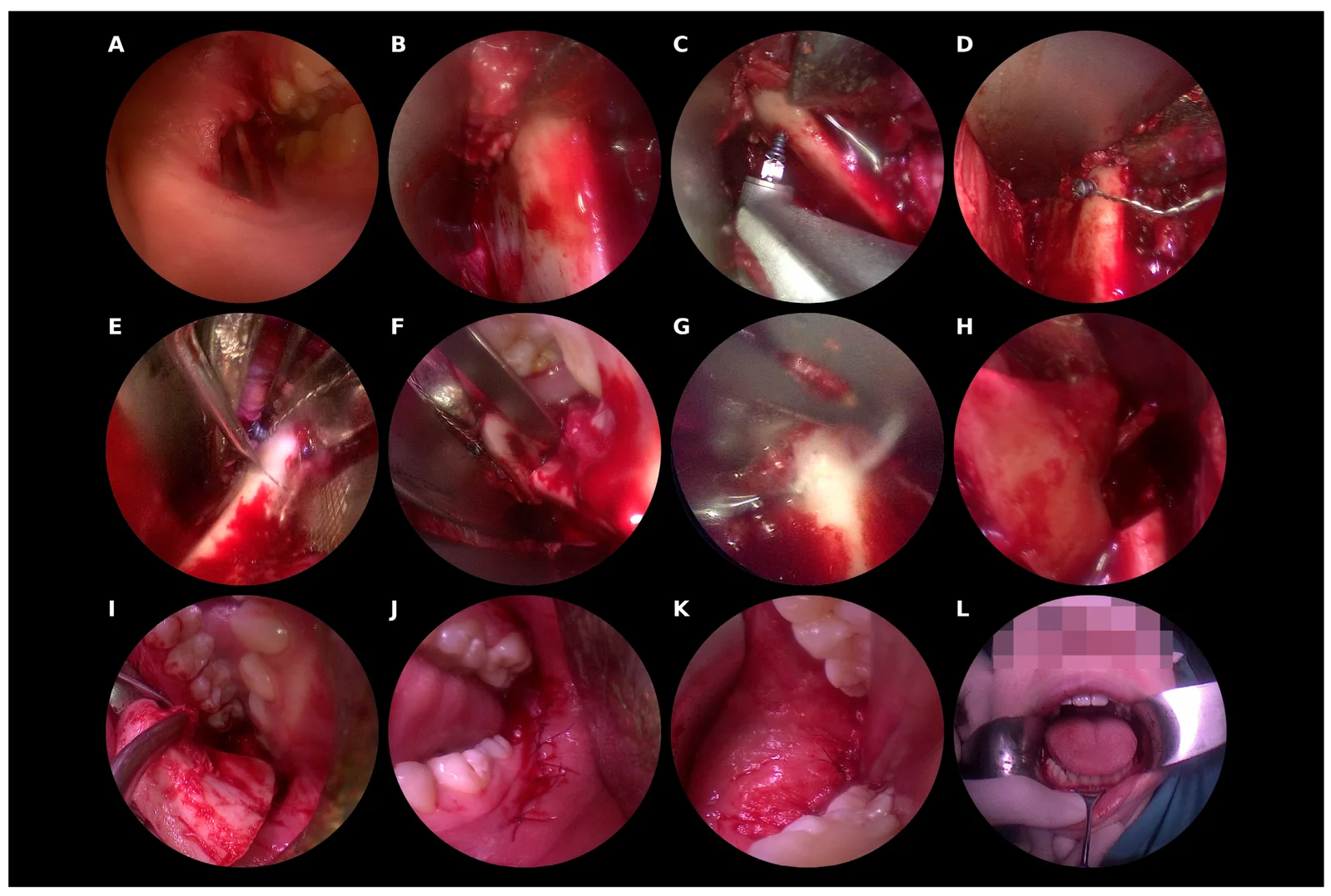
Endoscopically assisted intraoral bilateral coronoidectomy at the age of 18. (**A**) Intraoral view of the incision line along the right external oblique ridge, from the first molar region superiorly; the entrance to the subperiosteal tunnel on the lateral surface of the ramus is seen. (**B**) Endoscopic view inside the tunnel: the exposed anterior border of the coronoid process after subperiosteal dissection towards the sigmoid notch. (**C**) A self-drilling screw being inserted into the coronoid process with a 90° angulated screwdriver to gain control of the fragment before osteotomy. (**D**) The seated screw in the coronoid process with the twisted retention wire attached. (**E**) Osteotomy of the coronoid base with the piezoelectric surgical tip, directed from the anterior border of the ramus towards the sigmoid notch. (**F**) Retrieval of the right coronoid process. (**G**) Osteotomy of the left coronoid base with the piezoelectric surgical tip. (**H**) Mobilized left coronoid process during sharp dissection of the tendon. The residual osteotomy surface of the ramus stump and the dead space towards the condyle are visible. (**I**) Retrieval of the resected left coronoid. (**J**,**K**) Bilateral wound closure with interrupted Glycolon 5-0 sutures. (**L**) Forced mouth opening at the end of the procedure, demonstrating immediate improvement (patient’s face masked for anonymity).

The patient underwent a bilateral endoscopically assisted coronoidectomy at the Department of Maxillofacial and Oral Surgery at Dubrava University Hospital (Zagreb, Croatia). After general anesthesia with nasotracheal intubation, a diluted local anesthetic with vasoconstrictor was administered to the oral surgical region: the mandibular angle, ramus and the coronoid process. An intraoral incision was made at the right external oblique ridge, 5 mm beyond the mucogingival line, extending from the first molar region superiorly to the ramus. A full-thickness mucoperiosteal flap was raised to expose the medial and lateral surfaces of the ramus (Figure 4A). Further subperiosteal tunnelling allowed the endoscopically assisted approach to the coronoid process. For optimal visualization, a 4 mm diameter and 30° lens angle rigid endoscope (Karl Storz, Tuttlingen, Germany) was used. A small bone hook verified the location of the sigmoid notch, and the coronoid process was secured by a self-drilling screw delivered by a 90° angulated screwdriver and attached to a twisted wire (Figure 4B–D). Osteotomy was performed using a piezoelectric surgical knife, with chisel luxation for final separation (Figure 4E). The coronoid process was sharply dissected from the temporal muscle tendon, whereas a more difficult, blunt separation from the posterior aspect of the zygomatic bone was noted. The resected coronoid process was then retrieved through the incision (Figure 4F). A comparable surgical procedure was carried out on the left side of the mandible (Figure 4G–I). Wound closure on both sides was performed using a Glycolon 5-0 monofilament (polyglycolic acid and ε-caprolactone) absorbable suture (Advanced Medical Solutions GmbH, Nuremberg, Germany) (Figure 4J,K). Forced mouth opening at the end of the procedure improved to 35 mm (Figure 4L).

**Figure 5:**
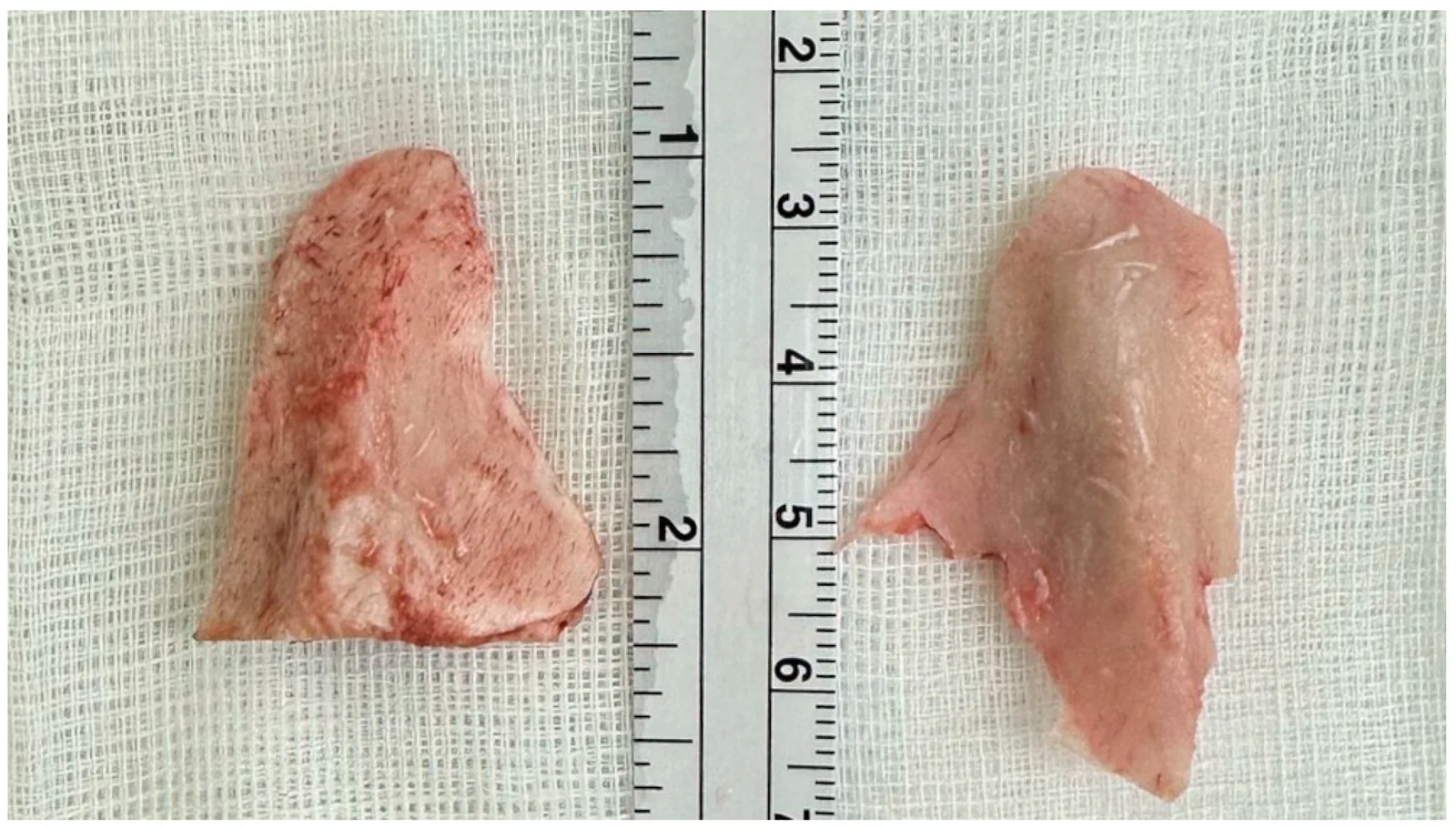
Resected coronoid processes on gauze with a centimetre ruler for scale, before histopathological examination (left side–right coronoid process; right side–left coronoid process).

The coronoid processes were subjected to histopathological examination (Figure 5), which showed thickened cortical bone with elements of bone marrow.

**Figure 6:**
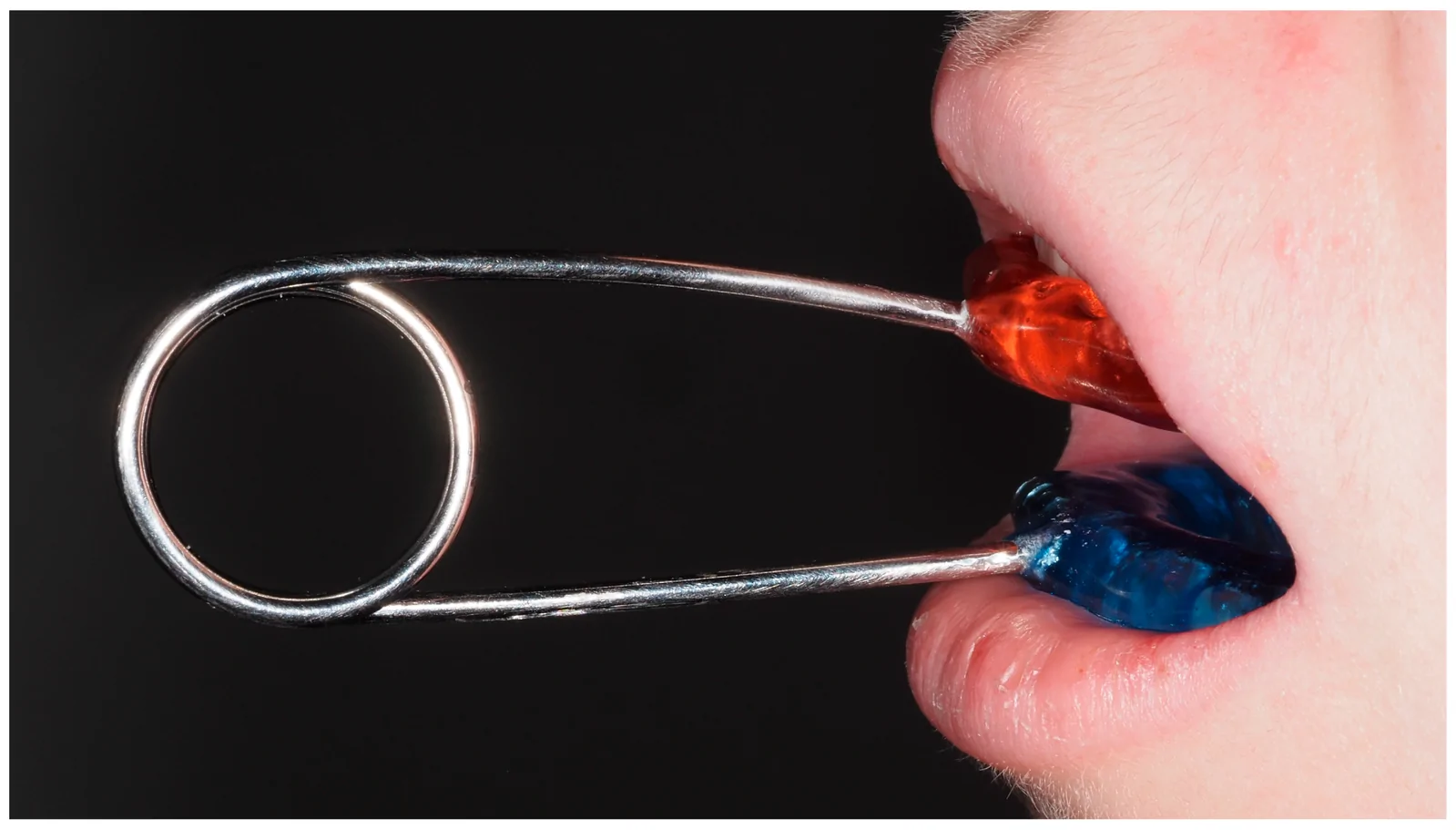
Miše small mouth opener in place on the fifth postoperative day (profile view): a spring-loaded stainless-steel wire device with acrylic occlusal pads seated on the opposing arches.

There were no complications during the surgery and the postoperative period was uneventful, with appropriate wound healing. During hospitalization, the patient received antibiotic and analgesic therapy with fluid and electrolyte replacement, and the postoperative hematomas regressed with regular wound care. Before discharge on the fifth postoperative day, a control orthopantomogram was unremarkable; the mandible was firm, the occlusion undisturbed, movements in both temporomandibular joints were clinically normal, and there was no sensory or motor deficit in the facial region. Discharge recommendations included rest, external cold compresses, head elevation, a soft diet with avoidance of hot and spicy food, and regular oral hygiene with sage-tea rinses after meals. Discharge medication comprised oral amoxicillin–clavulanic acid twice daily and metronidazole 400 mg three times daily for four further days, analgesics as needed, diazepam 2 mg at bedtime and an oral vitamin B complex; if required, an analgesic could be taken 30 min before the mouth-opening exercises. This regimen should be taken into account when interpreting the early postoperative pain scores. A Miše small mouth opener was fabricated and provided to the patient on the fifth postoperative day (Figure 6), to be used interchangeably with wooden spatulae for at least three exercise sessions daily, each lasting 15 min. Stretching was performed to patient tolerance, without quantification of the opening force, and was supported by local heat application. The device-based regimen was maintained through the first postoperative month, and the exercise techniques were reviewed at scheduled outpatient visits (two weeks; one, two, three, six and nine months, one year after surgery); no additional rehabilitation measures were required during follow-up. Adherence to the exercise protocol was not formally assessed; the patient’s daily completion of the electronic questionnaires during the first postoperative week was the only indirect indication that she followed the instructions.

**Figure 7:**
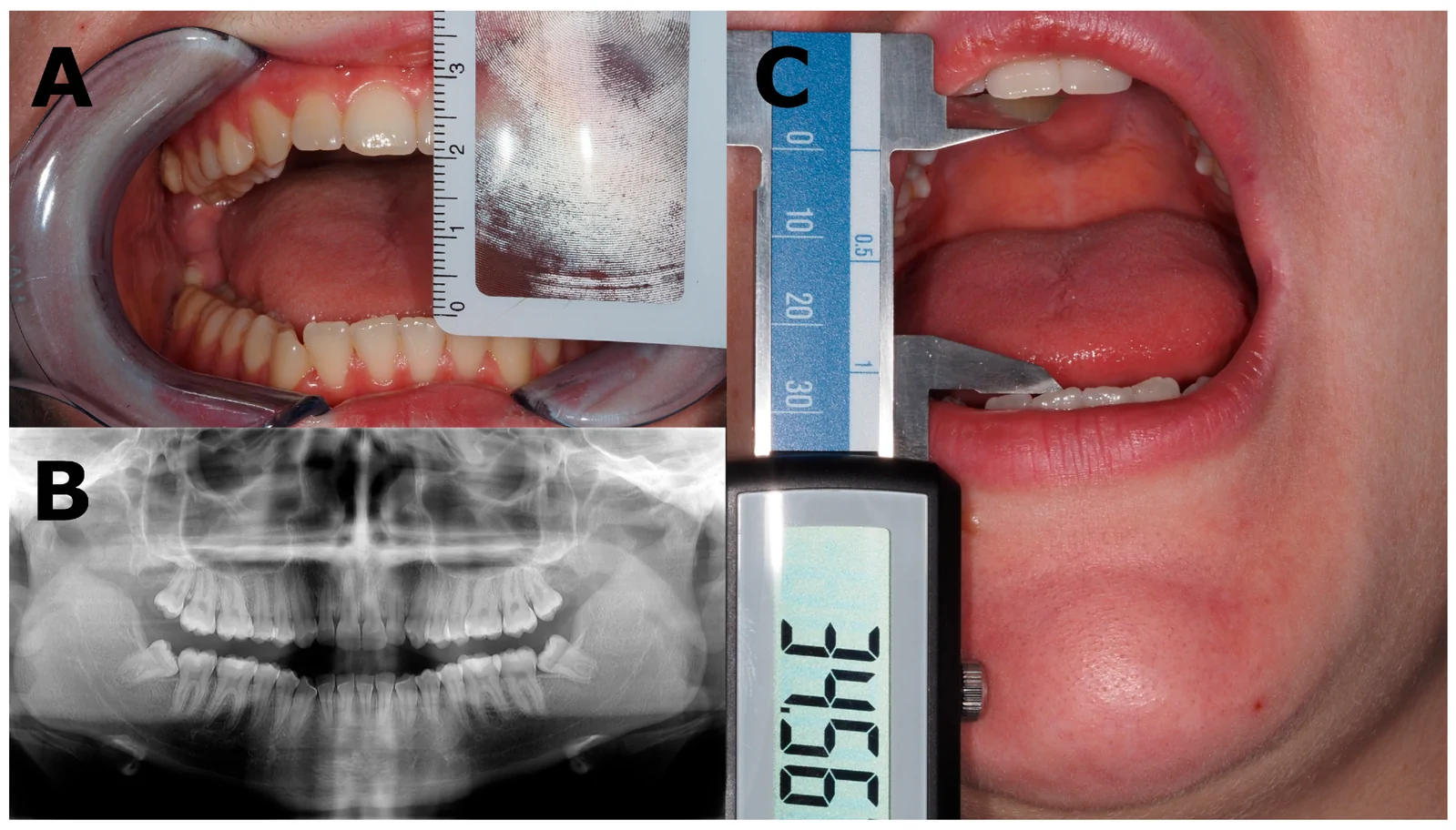
Postoperative findings. (**A**) Maximal unassisted interincisal opening of 20 mm at one month, measured with a millimetre ruler; (**B**) panoramic radiograph at 6 months showing the bilaterally resected coronoid processes with smooth remodelling of the anterior borders of the rami; (**C**) maximal unassisted opening at 9 months, measured with a digital calliper reading 34.56 mm, corresponding to recovery to a functional mouth-opening range.

At one month postoperatively, the patient demonstrated a 20 mm unassisted mouth opening (Figure 7A), at which point isometric temporomandibular joint exercises were introduced to the patient for long-term muscle conditioning (three sessions daily, five minutes each). By six months, the patient had achieved a full unassisted mouth opening. A follow-up panoramic X-ray was taken 6 months postoperatively (Figure 7B). At 9 months, unassisted MMO was 35 mm (digital calliper reading 34.56 mm), indicating recovery to a functional mouth-opening range (Figure 7C). At the one-year recall, unassisted MMO measured 35 mm, remaining stable between nine months and one year; lateral and protrusive excursions were not numerically recorded at the follow-up assessments.

### 2.3. Quality-of-Life Outcome Assessment

Oral-health-related quality of life outcomes data were collected from the patient preoperatively, daily during the first seven postoperative days, and at one year by using an Oral Health Impact Profile 14 (OHIP-CRO14) questionnaire [20,21] and visual analogue scale (VAS), utilizing the Qualtrics XM Platform (Qualtrics, Provo, UT, USA).

The OHIP-14 questionnaire comprised 14 questions regarding quality-of-life impact covering seven domains on limitations, pain, discomfort and disability. Each question was rated on a 5-point Likert scale (0—never, 4—very often), where higher scores indicated greater negative impact [21]. Pain intensity was assessed using an electronic VAS [22], where the patient adjusted the slider positioned between “no pain” and “maximum pain”. The scale contained no gridlines or visible numeric indicators. Daily ratings were captured as values ranging from 0 to 100. Data were processed and visualized by using R statistical software (Version 4.6.0, R Core Team, Foundation for Statistical Computing, Vienna, Austria, 2026).

**Figure 8:**
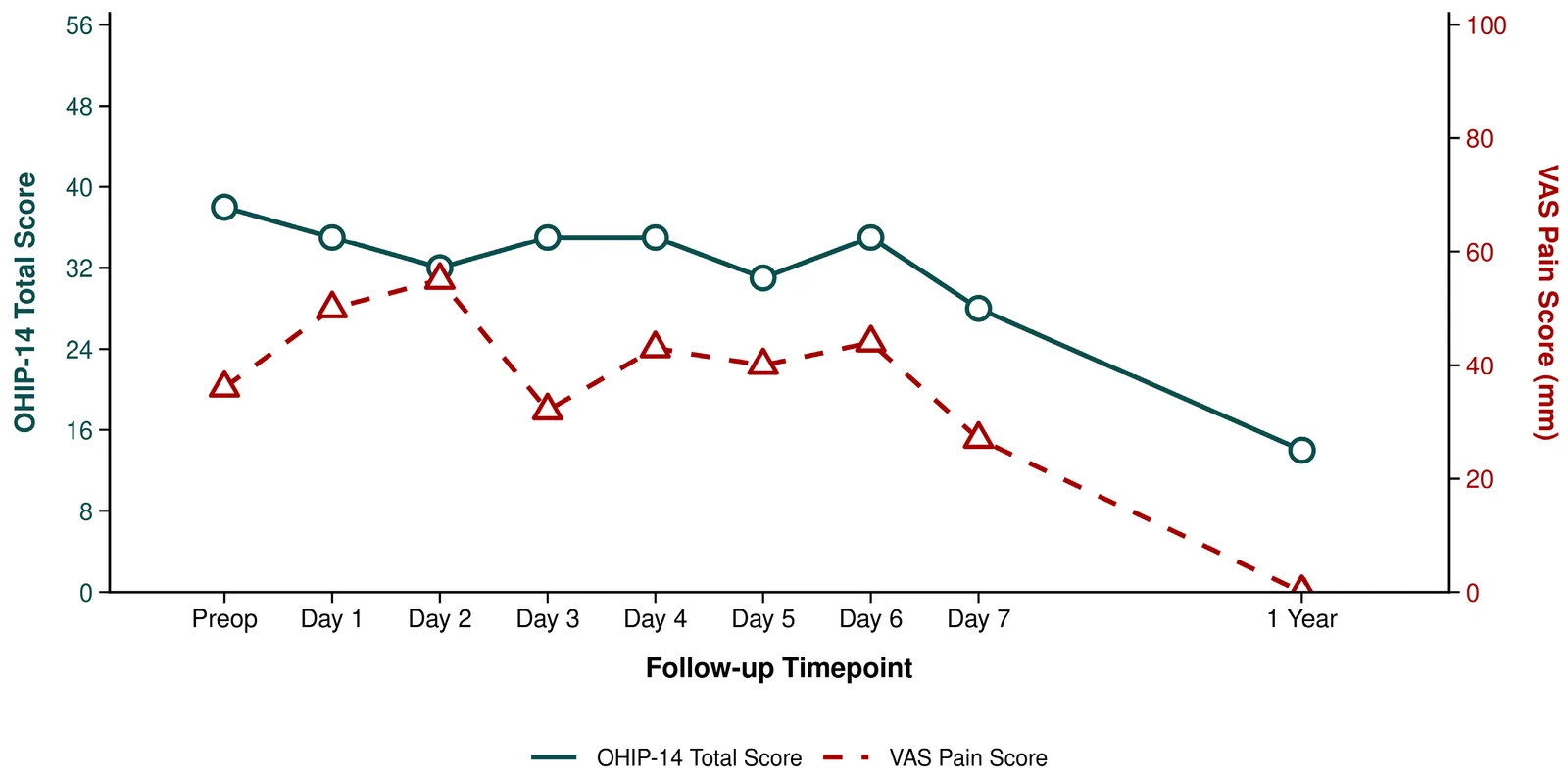
Oral Health Impact Profile-14 (OHIP-14) total score (solid line, left axis, range 0–56) and visual analogue scale (VAS) pain score (dashed line, right axis, 0–100 mm) preoperatively, on postoperative days 1–7, and at the 1-year recall. Higher scores indicate greater oral-health impact and greater pain, respectively.

Pain assessment scores rose sharply from a preoperative value of 36 to 55 in the first two postoperative days, crossing the moderate pain threshold [23], while the subsequent values fluctuated within the mild pain range (scores 27–44) up to day 7. At one year recall, there was no pain reported (Figure 8, dashed line).

**Figure 9:**
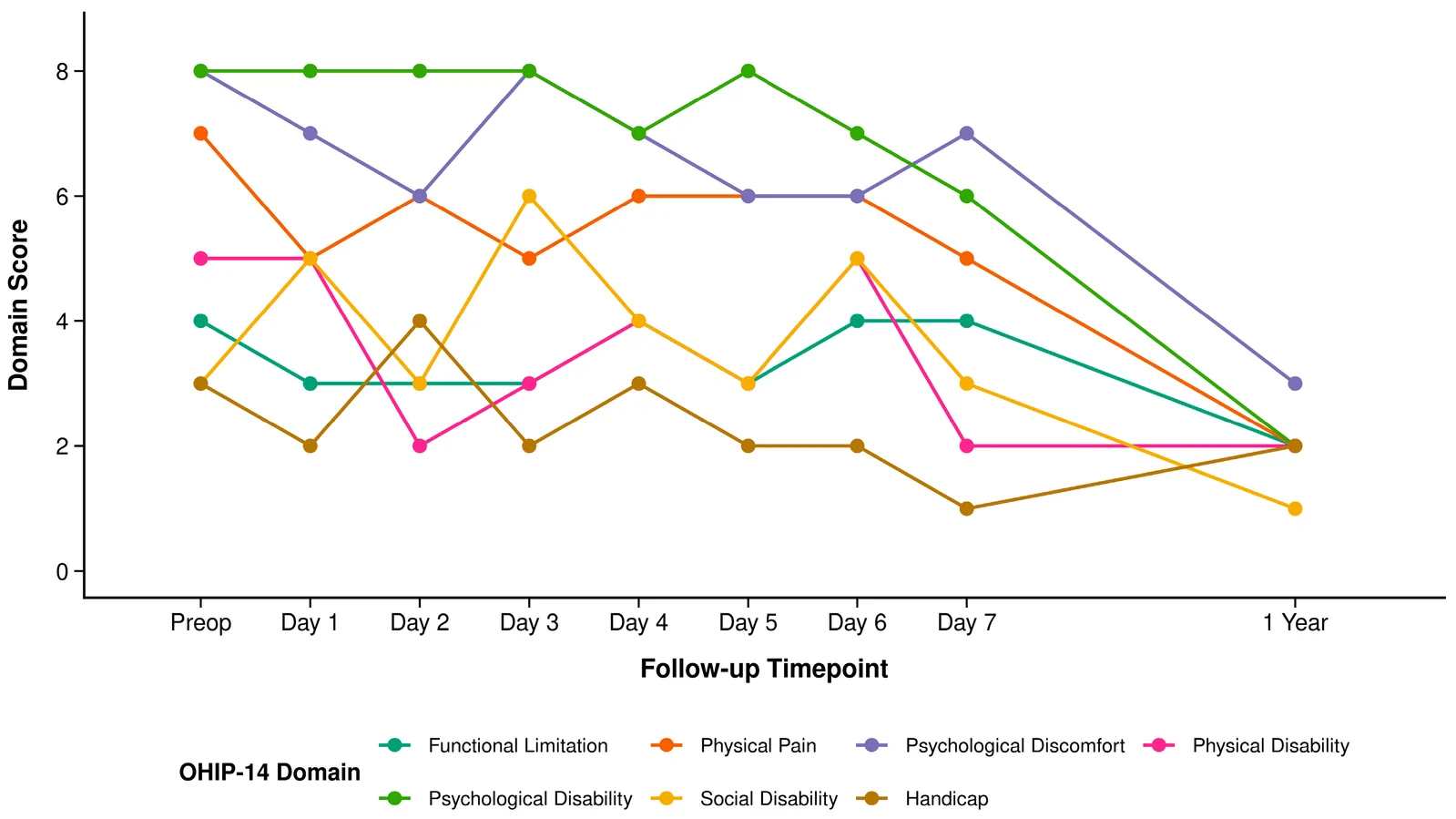
OHIP-14 domain subscale scores (seven domains, each 0–8: functional limitation, physical pain, psychological discomfort, physical disability, psychological disability, social disability, handicap) preoperatively, on postoperative days 1–7, and at the 1-year recall.

The OHRQoL assessment total score remained consistent with a poor-to-moderate impact [24] and low quality of life [25] from the preoperative point (score 38), throughout the course of the postoperative week (values 31–38), and had a sharp decline at day 7 (Figure 8, solid line). One year later, the total score decreased to 14, corresponding to good impact and very high quality of life. In the subscale scores for the seven domains involved, psychological disability and psychological discomfort showed the highest values (6–8) at all points, except for the 1-year recall, where most of the domains converged to the values 2–3 (Figure 9).

Physical pain values grouped with the psychological domains, whereas functional limitation and handicap were less pronounced. Social disability had the lowest initial and end values, oscillating the most in the immediate postoperative period.

## 3. Discussion

Coronoid hyperplasia is considered a rare underlying cause of restricted mouth opening, accounting for less than 5% of such cases, with unclear etiology [4,26]. Characterized by progressive limitation, it is mostly found bilaterally, showing a male-to-female ratio of 5:1, mean onset age of 14.7 years, and a mean preoperative MMO of 16.5 mm, as per the results of a review by Goh et al. [5]. In the literature analysis of Nogueira et al. [27], the lowest preoperative MMO reported was 9.5 mm. We presented a case of a 17-year-old female with bilateral coronoid hyperplasia, with the limitation onset age of 12, preoperative MMO of 8 mm, and surgical management upon turning the age of 18. The elongated coronoid process in question can present in different shapes. Nirala et al. [28] investigated reports on its morphological variations. The triangular form was most commonly reported, with the hook-shaped process reported as the second most prevalent variant in many studies and the rounded form among the least commonly reported. Our case exhibited the rounded variant. All these parameters fell below the reported means, underscoring the rarity of this presentation.

A tentative working diagnosis of JD was based on the early MSCT, preoperative CBCT findings and on the difficult intraoperative separation of the coronoid processes from malar contact. The definition of JD itself remains debated. Commenting on the classification proposal of Raccampo et al. [1], Mattei et al. [10] argued that JD denoted a pseudo-joint between the zygomatic bone and the coronoid process regardless of the condition of the process, and that histopathological proof of cartilaginous tissue on both articulating surfaces is required to verify the diagnosis. In their reply, the original authors [11] pointed out the time-dependent nature of the cartilage layer, which develops through chronic impingement and mutual remodelling of the two bony surfaces. JD has also repeatedly been regarded as a developed complication of coronoid osteochondroma [1,29,30,31]. Because histology in our case showed only thickened cortical bone with elements of bone marrow, our diagnosis of JD remained provisional. Case reports without verification of a cartilage layer nevertheless exist [1,32]; in particular, Zhao et al. [33] established the diagnosis of JD with a histopathological finding similar to ours, demonstrating “hyperosteogeny” and no fibrous or cartilaginous elements.

Surgery was carried out via an intraoral approach in order to avoid scarring and sensory or motor damage to the face. This route, particularly with a sparing incision design, limits visibility of and access to the coronoid process. Therefore, we opted for the less invasive, endoscopically assisted approach. Published experience with endoscopically assisted coronoidectomy for coronoid process hyperplasia or Jacob’s disease is summarized in Table 2 and remains limited to six reports comprising ten patients [1,9,17,18,34,35]. None of these reports included a quantified patient-reported outcome measure. Coronoidectomy without endoscopic assistance has been reported in larger series: Domart et al. [36] treated 20 patients with Langenbeck or Jacob disease by coronoidectomy followed by physiotherapy or self-rehabilitation, and mouth opening improved from 19.2 ± 7.0 mm to 38.0 ± 7.6 mm immediately after surgery.

Yoshida [37] investigated the effect of mouth-opening retaining appliances after coronoidotomy (the sectioned process was left in situ), and was the single article reporting quantified pain during the mouth-opening training. The author compared VAS scores, reported on a 0–10 metric, in patients with and without a silicone mouth-opening retaining appliance inserted immediately after surgery; mouth-opening training with a Heister jaw opener, starting on postoperative day 3, was performed in both groups. The data presented significantly less pain in the intervention group on day 1 (5.7 ± 0.78 vs. 6.6 ± 0.89), day 3 (6.0 ± 0.86 vs. 7.0 ± 0.99) and day 7 (6.6 ± 0.8 vs. 7.4 ± 0.98), emphasizing the benefit of the mouth-opening retaining appliance. Our physical exercise started on the fifth postoperative day, using an individually fabricated, coil-based, small mouth opener. Because the mouth-opening training in that study began on the third postoperative day, its training days 1, 3 and 7 correspond to postoperative days 3, 5 and 9, respectively. Converted to a 0–100 scale, the reported scores were 57, 60 and 66 in the appliance group and 66, 70 and 74 in the control group, whereas our VAS scores between postoperative days 3 and 7 fluctuated within the mild-pain range (27–44), below the values reported for either group. To date, no other comparable pain-related data was available in the literature. Jensen et al. [23] determined the cutoffs for pain intensities in a 100 mm VAS rating, with the values from 5 for mild pain, 45 for moderate pain, and 75 for severe pain. The authors also suggested a 33% decrease in pain as meaningful. We have noted a pre-operative pain value of 36, corresponding to mild pain, and no pain at the one-year recall, supporting the favourable pain course of the applied surgical approach.

OHRQoL outcomes have been regularly reported in dentistry and oral and maxillofacial surgery, pertaining to tooth decay and loss [38,39], periodontal therapy [40], third-molar surgery [41], implant prosthetic rehabilitation [42,43,44], temporomandibular disorders [45], dentofacial deformity [46], orthognathic surgery [47], and head and neck cancer [48]. To verify the novelty of the present data, we searched PubMed/MEDLINE on 19 August 2026, without date or language restrictions. The search was limited to the disease side, so that no outcome term could exclude a relevant report. The string was (coronoidectomy OR coronoidotomy OR “coronoid hyperplasia” OR “coronoid process hyperplasia” OR “coronoid process hypertrophy” OR “coronoid process elongation” OR “elongated coronoid” OR “Jacob disease” OR “Jacob’s disease”), and it returned 801 records. All records were screened at title and abstract level, and those describing surgery of the mandibular coronoid process together with any patient-reported or quality-of-life data were examined further, in full text where this could be obtained. The search identified no publication reporting quantified oral-health-related quality-of-life outcomes after coronoidectomy. Quality of life was mentioned only narratively, without any instrument or score, most directly as a statement of subjective improvement and satisfaction in the cohort of Miaśkiewicz et al. [16]. The only quantified pain measure recorded during postoperative mouth-opening training after coronoid process surgery was the visual analogue scale rating reported by Yoshida [37]. One study did use a validated patient-reported instrument. Qiao et al. [49] recorded FACE-Q satisfaction scores in patients with elongated coronoid processes treated by partial coronoidectomy combined with reduction malarplasty. Those scales measure satisfaction with facial appearance rather than oral health, and the operation was performed for midface contouring, not for restricted mouth opening. To the best of the authors’ knowledge, therefore, no prior report has used the OHIP-14 or provided domain-resolved OHRQoL data after coronoidectomy. These are single-case values and should be interpreted with caution. In terms of determining OHIP score spans, Altay et al. [25] interpreted values 0–14 as “very high”, 15–28 as “high”, 29–42 as “low”, and 43–56 as “very low”. Moreover, Novianti and Sufiawati [24] outlined the following impact categories: scores 0–18 as “good”, 19–37 as “moderate”, and 38–56 as “poor”. Our preoperative OHIP value of 38 corresponded with low quality of life and fell within the “poor” impact category. Interestingly, the surgery did not add to the chronic OHRQoL burden, even on days 1 and 2. In comparison, VAS did peak at day 2, and then it declined. This can be explained by the fact that VAS measurements capture acute pain, as opposed to the chronic construct of OHIP. The postoperative week demonstrated OHIP values consistent with low quality of life and “poor”-to-“moderate” impact. At the one-year recall, the total score decreased to 14, corresponding to very high quality of life and the “good” category. The reported 63% decrease is larger than the one-year reduction reported after orthognathic surgery by Tuk et al. [50], and consistent in direction with the improvement described by Choi et al. [51].

Subscale analysis across the seven domains revealed the most pronounced values in psychological disability and psychological discomfort from the preoperative timepoint to the end of the first postoperative week. Because the preoperative scores in these domains already approached the ceiling, the true acute postoperative worsening may have been masked. The values for handicap, functional limitation and social disability were the least pronounced and remained relatively preserved, apart from the preoperative and one-year time points. At the one-year follow-up, all subscale scores converged to low values, with the largest absolute reduction in psychological domains and pain.

The overall reduction in OHIP-14 values was largely driven by the restoration of functional jaw opening. Preoperatively, the patient could not eat or brush her teeth properly and experienced chronic pain from forced jaw use, with measurable psychological sequelae. Our surgical procedure yielded MMO improvement from 8 mm to 35 mm, corresponding to mean end-values reported in the literature [6,52,53,54,55].

## 4. Conclusions

This case report provides three meaningful clinical observations in the endoscopically assisted management of bilateral coronoid hyperplasia with suspected (provisional) Jacob’s disease. First, the intraoral approach to coronoidectomy and physical therapy yielded a 27 mm gain (+338%) in maximum mouth opening, from a severely restricted 8 mm baseline to a functional 35 mm at nine months postoperatively, corresponding to recovery to a functional mouth-opening range. Second, oral-health-related quality of life outcomes, measured by OHIP-14, demonstrated poor-to-moderate impact and low quality of life from the preoperative point throughout the course of the postoperative week, with a sharp improvement on day 7. Psychological disability and psychological discomfort exhibited the greatest impact. An improvement of 63% was noted from the preoperative point to one year postoperatively, with the most pronounced recovery in the psychological domains. Third, the visual analogue scale pain score peaked on day 2, whereafter it correlated with OHIP scores, with pain resolving fully at one year postoperatively. To the best of the authors’ knowledge, this is the first case report of domain-resolved oral-health-related quality-of-life outcomes after coronoidectomy. The findings represent a single-case observation: they cannot be generalized or regarded as a definitive baseline, but they may help inform the selection of instruments and assessment time points in future studies of larger cohorts.

## Figures and Tables

**Table 1:** Clinical timeline of the presented case.

Age/Time Point	Event
Age 12	Onset of progressive, painless limitation of mouth opening; no history of trauma.
Age 13	MRI of the temporomandibular joints (no joint pathology); MSCT of the maxillofacial region: bilateral hyperplastic coronoid processes, tips extending 3 mm beyond the level of the zygomatic arch in the closed-mouth position, with signs of coronoid–zygomatic remodelling.
Age 14	Petite stature noted (45 kg/156 cm), mild right-sided facial asymmetry, subtle right eyelid ptosis, planovalgus foot, metatarsus adductus; genetic work-up for suspected trismus-pseudocamptodactyly syndrome (clinical exome sequencing including MYH8, and chromosomal microarray); no clinically significant variants associated with the referral diagnosis were identified.
Age 15	Physiatric examination for ankle tendon and joint pain, attributed to a growth spurt.
Age 17	First presentation to the authors’ oral surgery clinic; unassisted MMO 8 mm.
Shortly before turning 18	Preoperative CBCT: bilateral CPH with suspected fibrous coronoid–zygomatic adhesions; provisional Grade 3 Jacob’s disease.
Age 18	Hospital admission; bilateral endoscopically assisted intraoral coronoidectomy; forced mouth opening at the end of the procedure improved to 35 mm.
Postoperative days 1–7	Daily OHIP-CRO14 and VAS assessments (electronic questionnaires).
Postoperative day 5	Custom Miše coil small mouth opener delivered; used interchangeably with wooden spatulae in at least three exercise sessions daily (15 min each); hospital discharge on the same day.
Outpatient follow-up	Scheduled check-ups at 2 weeks and at 1, 2, 3, 6 and 9 months and 1 year postoperatively.
1 month	Unassisted MMO 20 mm; progression to isometric temporomandibular joint exercises (three sessions daily, five minutes each).
6 months	Full unassisted mouth opening on clinical inspection (no standardized calliper measurement); follow-up panoramic radiograph.
9 months	Unassisted MMO 35 mm.
1 year	OHIP-14 total score 14; VAS 0; unassisted MMO 35 mm.

MMO = maximum mouth opening; CPH = coronoid process hyperplasia; CBCT = cone-beam computed tomography.

**Table 2:** Published reports of endoscopically assisted coronoidectomy for coronoid process hyperplasia or Jacob’s disease.

Authors (Year) [Reference]	n	Age/Sex	Diagnosis	Approach and Technique	Pre-Op MMO	Post-Op MMO (Time Point)	Follow-Up
Mavili et al. (1999) [34]	1	17/M	Unilateral (right) CPH	Endoscopically assisted, trans-temporal (two short temporal scalp incisions)	NR	NR	NR
Robiony et al. (2012) [9]	1	NR/M	Bilateral CPH	Description of endoscopically assisted intraoral coronoidectomy	21.5 mm (figure reading)	29.5 mm (1 month) (figure reading)	1 month
Dell’Aversana Orabona et al. (2022) [18]	1	18/M	Bilateral CPH	Intraoral endoscopically assisted, CAD/CAM cutting guides	16 mm	48 mm (intraoperatively)	NR
Raccampo et al. (2023), case 3 [1]	1	23/M	Bilateral Jacob’s disease	Intraoral endoscopically assisted	20 mm	50 mm immediately (forced opening); 37 mm (14 months)	14 months
Fernández Sanromán et al. (2024) [17]	5	Mean 25 y; 3 M, 2 F	3× bilateral, 2× unilateral CPH	Intraoral endoscopically assisted, piezoelectric instruments	17.2 mm (median)	38 mm (median, at last follow-up)	12–170 months (median 72)
Pazziani et al. (2026) [35]	1	25/M	Unilateral (left) CPH	Intraoral endoscopically assisted, 30° endoscope, piezoelectric osteotomy	18 mm	28 mm (intraoperatively)	NR

CPH = coronoid process hyperplasia; MMO = maximum mouth opening; NR = not reported.

## Data Availability

The data supporting the findings of this case report are contained within the article. Underlying patient-level OHIP-14 and VAS records are not publicly available in order to protect patient confidentiality but are available from the corresponding author on reasonable request.

## Supplementary Materials

The following supporting information can be downloaded at https://www.mdpi.com/article/10.3390/jcm15176591/s1, Table S1: CARE Checklist of information to include when writing a case report.
