# Supplementary material for: Endoscopically Assisted Management of Bilateral Coronoid Hyperplasia: A Case Report with Oral-Health-Related Quality of Life Outcomes
Source: J Clin Med. 2026 Aug 26;15(17):6591. doi: 10.3390/jcm15176591 (PMC13565797; doi:10.3390/jcm15176591)
Supplement: Supplementary file 1 [file jcm-15-06591-s001.zip › jcm-4481317-supplementary.pdf]

# Table S1: CARE Checklist of information to include when writing a case report

Manuscript: Endoscopically Assisted Management of Bilateral Coronoid Hyperplasia: A Case Report with Oral Health-Related Quality of Life Outcomes

Authors: Juraj Brozović, Igor Čvrljević, Marko Tarle, Barbara Mikulić, Bruno Vidaković, Ivica Lukšić

Journal: Journal of Clinical Medicine (MDPI) — Case Report

| Topic | Item | Checklist item description | Reported on line(s) |
|---|---|---|---|
| **Title** | **1** | The diagnosis or intervention of primary focus followed by the words "case report" | Title, lines 2–4; article type designation, line 1 |
| **Key Words** | **2** | 2 to 5 key words that identify diagnoses or interventions in this case report, including "case report" | Lines 31–32: coronoid hyperplasia; trismus; endoscopically assisted coronoidectomy; oral health-related quality of life; case report. Five key words, identifying the diagnosis, the intervention and the reported outcome, and including "case report". This revised set replaces the six key words carried by the version first submitted and occupies the same lines 31–32. |
| **Abstract** | **3a** | Introduction: What is unique about this case and what does it add to the scientific literature? | Lines 12–14 and 28–30 |
| | **3b** | Main symptoms and/or important clinical findings | Lines 14–16 |
| | **3c** | The main diagnoses, therapeutic interventions, and outcomes | Lines 12–25 (diagnosis and intervention, lines 12–16; outcomes, lines 18–25) |
| | **3d** | Conclusion—What is the main "take-away" lesson(s) from this case? | Lines 25–30 |
| **Introduction** | **4** | One or two paragraphs summarizing why this case is unique (may include references) | Section 1, lines 33–84; rationale and novelty statement, lines 76–84 |
| **Patient Information** | **5a** | De-identified patient specific information | Section 2.1, lines 87–89. All patient information throughout the report is de-identified: no name, date of birth or medical record number appears; the clinical photographs are cropped to the perioral region, showing no facial features that would permit recognition; and the panoramic radiograph carries no burned-in identifiers. |
| | **5b** | Primary concerns and symptoms of the patient | Lines 87–90 |
| | **5c** | Medical, family, and psycho-social history including relevant genetic information | Medical and developmental history, lines 90–93; family history, lines 103–105; genetic testing (MYH8 gene panel, chromosomal microarray), lines 105–109; musculoskeletal assessment, lines 109–111; psycho-social impact, lines 88–90 and 305–307 |
| | **5d** | Relevant past interventions with outcomes | Lines 93–96 (previous MRI and MSCT imaging), lines 105–109 (genetic work-up, unrelated to the diagnosis) and lines 109–111 (physiatric assessment). No previous surgical or conservative treatment had been undertaken for the restricted mouth opening before referral. |
| **Clinical Findings** | **6** | Describe significant physical examination (PE) and important clinical findings | Section 2.2, lines 113–122; preoperative maximal interincisal opening of 8 mm, lines 113–115 (Figure 2, line 124) |
| **Timeline** | **7** | Historical and current information from this episode of care organized as a timeline | Chronology reported in narrative form: onset at age 12 and imaging at age 13, lines 87–98; family history and genetic work-up, ages 14–15, lines 103–111; presentation at age 17, lines 87 and 113–122; surgery at age 18, lines 128–151; postoperative day 5, lines 156–159; 1, 6 and 9 |

| | | | |
|---|---|---|---|
| | | | months, lines 163–167; schedule of the patient-reported assessments (preoperative, daily to day 7, and 1 year), lines 174–177.<br>The authors first met the patient when she was referred for surgical treatment at the age of 18. The course of the disorder before that point was outside the authors' influence and has been reconstructed from the patient's existing medical documentation; only the perioperative and follow-up intervals were determined by the treating team. |
| **Diagnostic Assessment** | **8a** | Diagnostic testing (such as PE, laboratory testing, imaging, surveys) | MRI and MSCT, lines 93–98 (Figure 1, line 100); genetic testing, lines 105–108; clinical examination, lines 113–119; preoperative CBCT, lines 119–122 (Figure 3, lines 126–127); histopathology, lines 152–153 (Figure 5, line 155); patient-reported instruments (OHIP-14, VAS), lines 174–186 |
| | **8b** | Diagnostic challenges (such as access to testing, financial, or cultural) | Diagnostic delay of approximately six years between symptom onset and surgical treatment, lines 87–89 and 213–215; the requirement to exclude the commoner intracapsular and extracapsular causes first, lines 60–66; undocumented family history, lines 103–105; limits on confirming Jacob's disease histologically, lines 232–244 |
| | **8c** | Diagnosis (including other diagnoses considered) | Bilateral coronoid process hyperplasia with provisional Grade 3 Jacob's disease, lines 119–122 and 221–225; histopathological findings, lines 152–153; alternative diagnoses considered—trismus-pseudocamptodactyly syndrome (excluded genetically), lines 105–109; temporomandibular disorders, fracture, osteoma and osteochondroma, lines 44–50 and 60–66; discussion of the differential and of why the Jacob's disease diagnosis remained provisional, lines 221–244 |
| | **8d** | Prognosis (such as staging in oncology) where applicable | Lines 163–167 (full functional recovery by 9 months) and lines 305–309; expected outcome benchmarked against published end values, line 309 |
| **Therapeutic Intervention** | **9a** | Types of therapeutic intervention (such as pharmacologic, surgical, preventive, self-care) | Bilateral endoscopically assisted intraoral coronoidectomy under general anaesthesia, lines 128–151; postoperative mouth-opening physiotherapy, lines 156–159 and 163–165 |
| | **9b** | Administration of therapeutic intervention (such as dosage, strength, duration) | Anaesthesia and local infiltration, lines 130–132; incision design and flap, lines 132–135; endoscope specification (4 mm, 30°), lines 136–138; osteotomy and fragment retrieval, lines 138–143; wound closure and suture material, lines 143–146; contralateral procedure, lines 146–147; physiotherapy regimen (small mouth opener and wooden spatulae, at least three sessions daily from postoperative day 5), lines 156–159 |
| | **9c** | Changes in therapeutic intervention (with rationale) | Lines 163–165: isometric temporomandibular joint exercises were added at one month, once 20 mm of unassisted opening had been achieved, for long-term muscle conditioning |
| **Follow-up and Outcomes** | **10a** | Clinician and patient-assessed outcomes (if available) | Clinician-assessed: intraoperative opening, lines 147–148; maximal unassisted opening of 20 mm at 1 month and 35 mm at 9 months, lines 163–167 (Figure 7, lines 170–171). Patient-assessed: OHIP- |

| | | | |
|---|---|---|---|
| | | | 14 and VAS, Section 2.3, lines 174–206 (Figures 8 and 9, lines 193 and 203); summarized lines 311–322 |
| | **10b** | Important follow-up diagnostic and other test results | Histopathological examination of the resected coronoid processes, lines 152–153; follow-up panoramic radiograph at 6 months, lines 165–166 (Figure 7b, lines 170–171) |
| | **10c** | Intervention adherence and tolerability (How was this assessed?) | Tolerability: uneventful postoperative course with appropriate wound healing, lines 156–157; pain during the exercise period quantified daily by VAS, lines 187–190. Adherence: the prescribed regimen is stated in lines 157–159 and the patient completed the electronic questionnaire on each of the seven postoperative days and at one year, lines 174–177 |
| | **10d** | Adverse and unanticipated events | Lines 156–157: no intraoperative complications and an uneventful postoperative course; no adverse or unanticipated events occurred |
| **Discussion** | **11a** | A scientific discussion of the strengths AND limitations associated with this case report | Strengths, lines 280–283 (first domain-resolved OHRQoL data after coronoidectomy, proposed as a baseline for future comparison). Limitations, lines 298–301 (possible ceiling effect masking acute worsening in the psychological domains) and lines 322–325 (single-case observation requiring evaluation in larger cohorts) |
| | **11b** | Discussion of the relevant medical literature with references | Section 3, lines 207–309, with 52 references (lines 359–487) |
| | **11c** | The scientific rationale for any conclusions (including assessment of possible causes) | Interpretation of the divergence between acute pain (VAS) and the chronic OHIP construct, lines 286–295; attribution of the OHIP-14 reduction to restored jaw function, lines 305–309; aetiological considerations for coronoid hyperplasia and Jacob's disease, lines 221–244 |
| | **11d** | The primary "take-away" lessons of this case report (without references) in a one paragraph conclusion | Section 4, lines 310–325 (no references cited) |
| **Patient Perspective** | **12** | The patient should share their perspective in one to two paragraphs on the treatment(s) they received | The patient's perspective was captured quantitatively rather than narratively, through her own daily OHIP-14 and VAS ratings reported in Section 2.3 (lines 174–206) and interpreted in lines 296–307. A narrative statement in the patient's own words is not included in the submitted version; it has been prepared and can be incorporated as a separate subsection at revision or supplied to the Editor on request. |
| **Informed Consent** | **13** | Did the patient give informed consent? Please provide if requested | Yes ☒ No ☐<br>Written informed consent for the procedure and for publication of all clinical data, photographs and imaging in anonymized form: lines 340–342. Ethics Committee approval (Dubrava University Hospital, ref. 2026/0521-4): lines 337–339. The signed consent form is available to the Editor-in-Chief on request. |
